# Montana

**Published:** 1897-02

**Authors:** 


*Montana.*—A bill is pending before the present Legislature providing for the inspection of meat and milk. Senator Metzell, who introduced the bill, urges strongly its adoption in an interview in the *Daily Inter-Mountain*, of Butte City. He forcibly presents the many dangers and deceptions incidental to the unguarded sale of these products. The bill provides for the appointment of inspectors, who must be veterinarians and graduates of colleges recognized by the United States Veterinary Medical Association. No meat or milk can be sold or given away without the approval of inspectors. Every cow furnishing milk for public sale must be tested with tuberculin and a certificate given for each individual cow.

A bill will also be presented looking to the protection of the interests of veterinarians in Montana.

